# Developmental trajectory of rule management system in children

**DOI:** 10.1038/s41598-018-31235-6

**Published:** 2018-08-24

**Authors:** Taeko Harada, Motoharu Tsuruno, Tetsuya Shirokawa

**Affiliations:** 1 Research Center for Child Mental Development, Hamamatsu University School of Medicine, Handayama 1-20-1, Hamamatsu, Shizuoka 431-3192 Japan; 2 Mebae Child Development Academy, Minamicho1-26 2F, Saidaiji, Nara, 631-0824 Japan; 3 Faculty of Sport Sciences, Nihon Fukushi University, Okuda, Mihama, Aichi 470-3295 Japan

## Abstract

The ability to apply rules for environmental adaptation is crucial for human life. This capacity may require high-order cognitive control, such as when managing personal behavior by selecting among context-dependent internal rules. This process is poorly understood in children, especially in terms of the age at which multiple-rules processing becomes possible. We created a child-appropriate “rule management paradigm” to elucidate developmental changes in rule processing, and used it to investigate the trajectory of the rule management system in 322 children aged 4 to 6 years, with comparison to 57 adults. We found age-specific capacities in multiple-rules processing, with the majority of 4-year-olds failing at concurrent management of multiple-rules processing, a capacity that became well developed by age 6. Task performance in multiple-rules processing improved steeply with age and approached the adult level by late age 6. By contrast, single-rule processing on single-feature stimuli approached the adult level by age 5. Our main findings suggest that the critical period for the development of the multiple-rules processing system occurs before age 7, and is associated with the developmental period of the rule management system and other cognitive resources.

## Introduction

The ability to manage responses to context-dependent internal rules is crucial to human development and survival. The ability to select among rules according to changing conditions and to convert decisions into a series of actions is a complex function within the hierarchy of higher executive control. However, the nature of the activation and development of this process during childhood has yet to be elucidated. Young children show difficulty in managing multiple-rules processing, which represents a predictable functional immaturity. They cannot select and apply proper rules according to the situation and show difficulty managing rule changes (e.g., the salience of color versus shape^1^). To elucidate the nature and developmental trajectory of this capacity, we must identify the age-related dynamic changes that may form the foundation of later, complex, higher-level constructs subserving the hierarchical organization of the human brain. Further, it would be useful to identify the basis in neural development by which rule manipulation varies with stimulus presentation in children.

The developmental latency of executive functions (EFs) tracks with the capacity for rule management in children. Theories of EFs and their development have emphasized the importance of cognitive complexity^2,3^, such that children develop the capacity to understand increasingly complex relations among objects and can do so in parallel^4^. The Cognitive Complexity and Control (CCC) theory hypothesizes that the development of EFs can be understood in terms of age-related increases in the maximum complexity of the rules^5^. This CCC theory has been tested by analyzing children’s performance on hierarchical rules, using the Dimensional Change Card Sort (DCCS) task^5^. In this task, children must reflect on two sets of rules and construct a representation of a rule structure that can integrate the rules for different feature dimensions of a stimulus. It has been reported that this process can be managed by children by age 5^6–8^. However, this task is mainly applied to younger children’s rule systems to examine set-shifting or inhibitory control by changing between rule sets, but it is incapable of elucidating the development of integrative processing of rules, such as concurrent processing for multiple rules responding to stimuli at a higher order of EF. Further, it does not elucidate the transition period from single-rule to multiple rules-management processing in the development of the hierarchical rule system. Overall, the age-related developmental trajectory of the ability to manage a rule hierarchy is poorly understood. Thus, the present study focused on the period in which children develop the ability to manage multiple rules to better elucidate the development of the rules management system.

According to the developmental trajectories of cognitive function, core EFs (i.e., cognitive flexibility, inhibition, and working memory^9^) develop markedly between ages 3 and 5^10,11^, with some variation in developmental timeframes^12–15^. The construction of the foundational components is essential for the development of higher cognitive processes^12^. Specifically, it suggested that single-EF component develop in the initial preschool years, as well as the unity of all EF components, occurs between ages 5 and 9^16–18^, and that their functional differentiation occurs after about age 9^19–21^. Further, it has been suggested that the development of EFs reflects a more qualitative change in cognitive function between ages 3 and 5, whereas later developments reflect quantitative refinements and enhancements^22^. Thus, it appears that multiple rules processing, which reflects the capacity for rule integration, follows the formation of more basic EF components, typically between ages 5 and 6. It is during this period that transition takes place in the hierarchical structure of rule systems in children.

Complex rule management involving multiple EF components has been investigated using the Wisconsin Card Sorting Test (WCST), the Tower of London (TOL), and dual-tasks in children aged 7 to young adults^9,16,23^. The WCST and TOL are, however, trial-based rule-learning tasks and as such do not involve explicit rule management processing. They are also not ideal for application in young children where they might obscure the origin of errors, in the case of the WCST^24^, or the problem-solving strategy applied, in the case of the TOL^14^. Further, the complexity of these tasks make it difficult to isolate specific cognitive processes, because each task engages a variety of executive processes^9,23,25,26^. However, a dual-task paradigm is capable of elucidating concurrent rule management processing since it requires the application of two distinct rules according to specific features of the presented stimuli^27–29^. However, dual tasks can be also complex, even for adults, and most previous dual-task paradigm studies have required a combination of sensory, motor, and cognitive processing, concurrently^30–34^. No previous study has investigated age-related development of the rule management system for higher-level processing in children. Therefore, we identified the need for a novel task paradigm, that was simplified and appropriate for young children, to assess the development of the multiple-rules processing system.

We developed a paradigm that enables the application of hierarchically different rules by comparing task performance. We are especially interested in the period during which children develop their processing capacity to elucidate the basic formation of integrative capacities in the executive system based on rule control processing. Therefore, we designed a rule management paradigm comprising four stages of conditions with various rule processing requirements. Our paradigm employs two-feature stimuli in a step-by-step manner as follows: the first condition requires single rule use corresponding to each single-feature stimulus (i.e., color or shape); the second condition requires alternate use of a single rule corresponding to each of two stimuli features (i.e., color or shape); the third condition requires single rule use in the context of conflict between two rules corresponding to two merged stimuli features (i.e., colored shape); and the fourth condition requires the concurrent management of two rules comprising a main rule, which is identical to that in the third condition, and a secondary rule, which is an additional rule, in response to both of two stimuli features. We administered these tasks to children ages 4 to 6 years, and to young adults ages 19 to 21 years. We hypothesized that the critical period for the formation of the multiple rules-management system occurs between ages 5 and 6.

## Results

### Subject characteristics

All subject groups were evaluated using a chi-square test and a one-factor ANOVA, as shown in Table 1. There was no significant group difference in sex or IQ, respectively: X^2^ (6) = 7.04, *p* = 0.317 and (*F*_6,372_ = 1.76, *p* = 0.157).

**Table 1:** Demographic information on groups of subjects.

Group	No. of Subjects			Handedness (N)			Months		IQ	
	Total	Male	Female	Right	Left	Ambidex rous	Mean	SD	Mean	SD
early age 4 (4.0–4.5 years)	59	30	29	43	9	7	51	1.7	106.15	12.32
late age 4 (4.6–4.11 years)	67	35	32	51	11	5	56	1.8	109.88	12.72
early age 5 (5.0–5.5 years)	62	32	30	53	7	2	62	1.6	106.15	12.32
late age 5 (5.6–5.11 years)	59	32	27	51	6	2	68	2.2	109.88	12.72
early age 6 (6.0–6.5 years)	38	20	18	34	4	0	74	2.2	109.74	13.09
late age 6 (6.6–6.11 years)	37	22	15	35	2	0	81	2	111.89	13.67
adult (19 ± 0.5 years)	57	35	22	51	5	2	N.A.	N.A	N.A.	N.A.

#### Age-related emergence of multiple-rules processing

We first evaluated the age-related emergence of multiple-rules processing by observing whether the main and secondary rule could be concurrently managed according to the stimuli. Table 2 shows the percentage of subjects who failed multiple-rules management in each group. Failure at multiple-rules processing was concluded when subjects were unable to apply both the main and secondary rule properly. In most cases, such failures appeared to reflect that application of just a single rule. In failed trials, the correct performance rates in applying either the main or secondary rule was 0%. Concurrent rule processing failure was common in children at early age 4, at which point more than half of the subjects failed. One third of late age 4 subjects also failed. Thereafter, this failure pattern steeply declined until late age 5, and no such pattern was observed from age 6 onward. In each case, we previously confirmed the subject’s understanding of the task rules (rules knowledge) by checking their responses to verbal answers and a button press at least three times. Hence, this failure likely reflects their capacity to manipulate multiple rules and/or their capacity to convert such manipulations into appropriate behavior.

**Table 2:** Failure rates in multiple-rules condition for each group.

Group	Failure Rate (%)
early age 4	50.85
(4.0–4.5 years)	
late age 4	31.43
(4.6–4.11 years)	
early age 5	11.94
(5.0–5.5 years)	
late age 5	5.08
(5.6–5.11 years)	
early age 6	0
(6.0–6.5 years)	
late age 6	0
(6.6–6.11 years)	
adult	0
(19 ± 0.5 years)	

### Task performance

Before analyzing the effect of age on task performance, we verified the influence of the trial order (1^st^ and 2^nd^) and rule features (color and figure) comprising the conditions designated as Simple, Conflict, and Multiple in each age group, using a Mann-Whitney U test. We confirmed that there were no effects of trial order (p > 0.13) or task characteristics in any age group (p > 0.17). All task performances were analyzed to assess the age-related development of rule management capacity using a one-way multivariate analysis of variance (one-way MANOVA) by comparing age group as the independent variable and task performance as the dependent variable.

**Figure 1:**
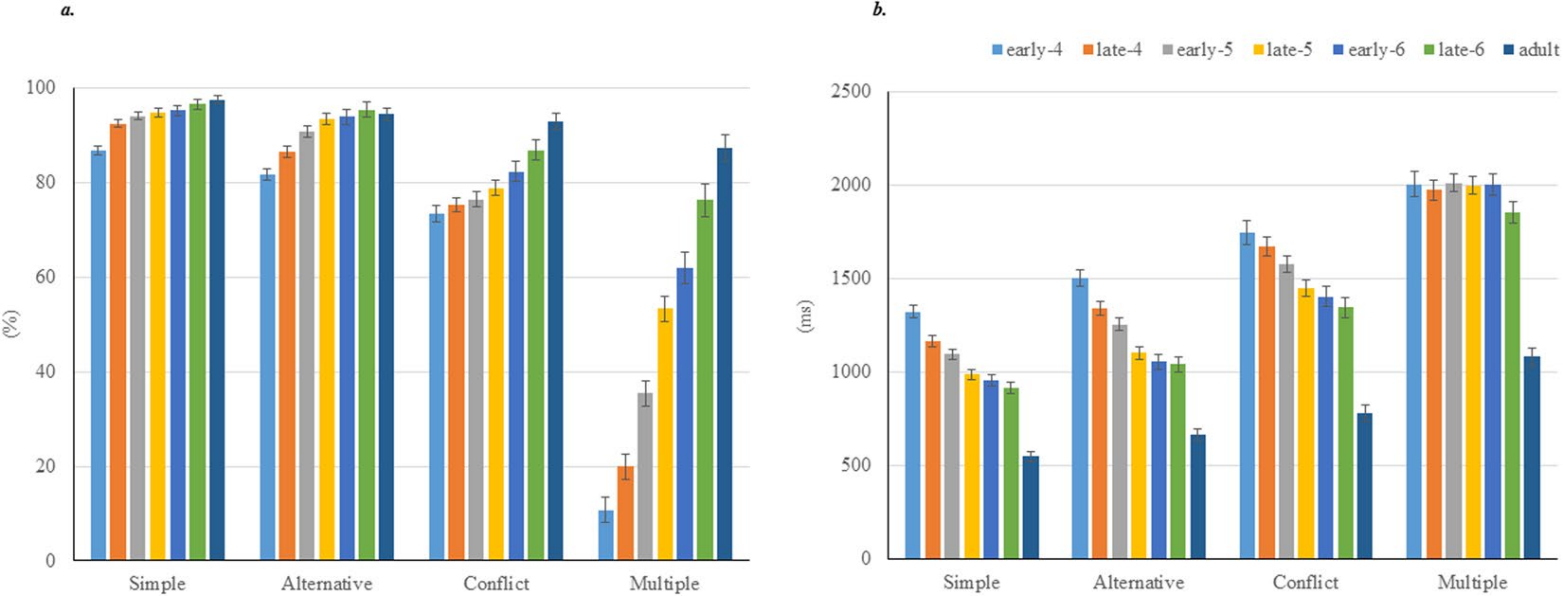
Task performance in four rule conditions in each age group. (**a**) Correct performance rates (%) and (**b**). Reaction times (ms) in the Simple, Alternative, Conflict conditions, and correct performance rates (%) calculated by harmonic mean in the Multiple condition in six groups of children (early age 4, early-4; late age 4, late-4; early age 5, early-5; late age 5, late-5; early age 6, early-6; late age 6, late-6) and one group of adults. Error bars represent standard errors.

As shown in Fig. 1a, there were significant main effects for age, *F* (24, 1288) = 19.62, p < 0.0001; Wilk’s Λ = 0.337, partial η^2^ = 0.238.

Additional findings were as follows:

- *Post-hoc* comparisons showed significant differences in performance in the Simple condition between early age 4 and all other age groups (p < 0.001), but no significant difference between early age 5 (p = 1.0) and all later ages. Compared with the adults, a significant difference was present until late age 4 (p < 0.002), but disappeared by early age 5 (p > 0.2).
- In the Alternative condition, there was a significant difference between early age 4 and all age groups above age 5 (p < 0.001), but no significant difference was observed from early age 5 thereafter (p > 0.6). Compared with the adults, there was a significant difference until late age 4 (p < 0.001), but this disappeared by early age 5 (p > 0.9).
- Regarding performance in the Conflict condition, there was no significant difference between early age 4 and early age 5 (p > 0.6), or between late age 5 and any age thereafter (p > 0.07). Compared with the adults, there was a significant difference until early age 6 (p < 0.003), but this disappeared by late age 6 (p = 0.608).
- In the Multiple condition, there was a significant difference between early age 4 and all groups older than age 5 (p < 0.001), but this difference disappeared by early age 6 (p = 0.073). Compared with the adults, there was a significant difference until early age 6 (p < 0.001), but this disappeared by late age 6 (p = 0.283).

Overall, lower performance levels were observed at both early and late age 4 in the Simple, Alternative, and Multiple conditions, but low performance persisted until early age 5 in the Conflict condition. No significant differences in performance were observed after early age 5 in the Simple and Alternative conditions, after late age 5 in the Conflict condition, or after age 6 in the Multiple condition. Additionally, there were no significant differences between the adults and children after age 5 in the Simple and Alternative conditions, or after late age 6 in the Conflict and Multiple conditions. These results suggest that higher-order rule management processing becomes established by a bottom-up development process in children between ages 5 and 6.

### Reaction Times

Figure 1b shows the means of the median reaction time (RT) for correct responses. The RTs showed significant main effects for age, *F* (24, 1065) = 23.15, p < 0.0001; Wilk’s Λ = 0.231, partial η^2^ = 0.307.

- *Post-hoc* analyses showed that RT in the Simple condition was significantly different between early age 4 and all other age groups (p < 0.02), although no differences were observed between late age 4 and early age 5 (p = 1.0), or between late age 5 and late age 6.5 (P = 1). Compared with the adults, significant differences were observed for all age groups of children (p < 0.001).
- In the Alternative condition, RT was significantly different between late age 4 and all other age groups (p < 0.001), but no such differences were not observed between late age 5 and late age 6 (p = 1.0). Compared with the adults, significant differences were observed for all groups of children (p < 0.001).
- In the Conflict condition, RT was significantly different between early age 5 and all other age groups of children (p < 0.006), but no such differences were observed between late age 5 and late age 6 (p = 1.0). Compared with the adults, significant differences were observed for all groups of children (p < 0.001).
- In the Multiple condition, RT showed no significant differences among any of the groups of children (p = 1.0). Compared with the adults, significant differences were observed for all groups of children (p < 0.001).

There were significant differences in the RTs between the children and adults in every task condition. However, the RTs among the children groups showed no significant changes between late age 5 and late age 6 in the Simple, Alternative, and Conflict conditions, and there was no significant difference between any children’s group in the Multiple condition.

## Discussion

The present study investigated age-related development of the rule management system by studying multiple-rules processing in children ages 4 to 6 years old. Our results showed that multiple-rules processing specifically develops between ages 5 and 6, and that failure to process multiple rules is common up to age 4, but improves dramatically thereafter until age 6. Task performance in the multiple-rules condition approached that of adults by late age 6, while single-rule performance approached adult levels by age 5 (Simple and Alternative conditions) in cases where there was no conflict in the stimuli. Further, development of adult-like performance levels in the Conflict condition was observed during the same timeframe under the multiple-rules condition. Thus, our main findings reveal that the multiple-rules management system has not yet emerged by age 4, but develops dramatically thereafter until late age 6.

Previous studies using a multiple-rules paradigm showed age-related differences in dual task performance between ages 5 and 8^30,31^, with adult-like performance in children older than age 8^30,33^. Even children age 7 showed no differences compared with children age 9, or with young and old adults^35^. Further, successful WCST task performance is reported to arise by age 7^36^. Since the capacity to manage multiple rules does not undergo marked developmental change after age 7, the system enabling concurrent use of multiple rules is likely to be formed prior to age 7^35,37^. The present results in the Multiple condition showed adult-level capacity by late age 6, and no failure in multiple-rules processing beyond age 6. Therefore, it is likely that the critical period for the development of this system is age 6. The present findings are consistent with previous studies in that performance in the Multiple condition reaches the adult level by age 7.

**Figure 2:**
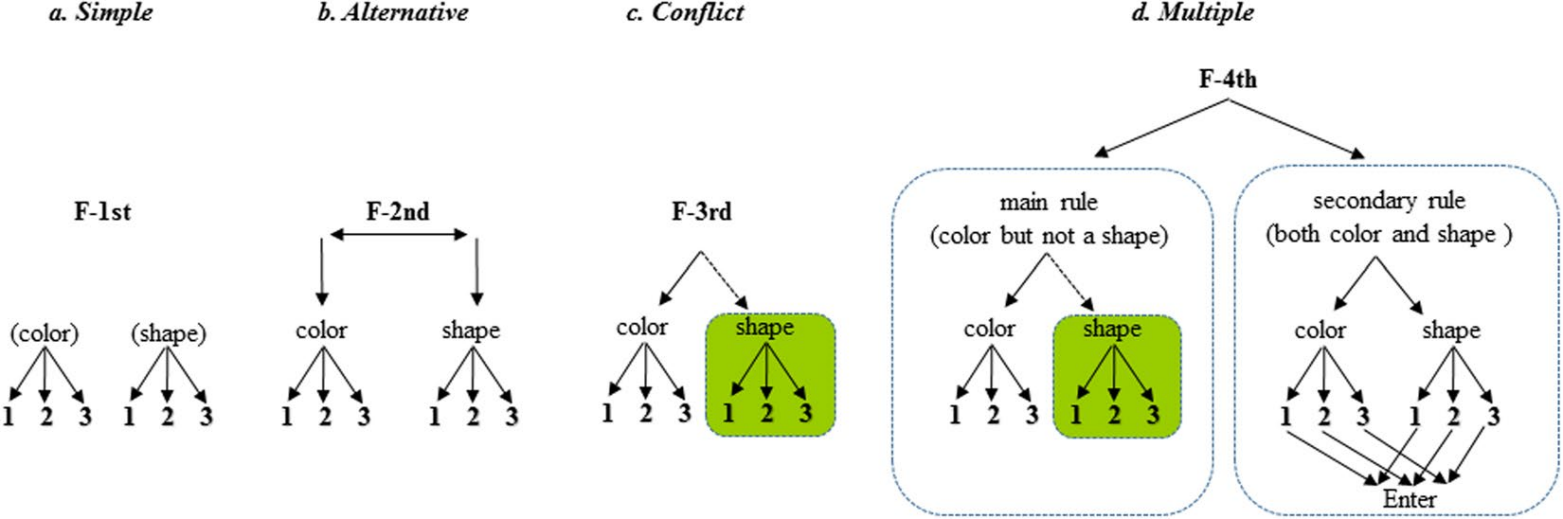
Hierarchical tree structure of rule systems. The syntactic tree depicts hierarchically different functions in each task rule, exhibited as F. It also depicts relations among rules and responses according to two-features stimuli (i.e., color and shape). (**a**) F-1st (Simple condition): single-rule operation is required for responding to each single-feature judgment. (**b**) F-2nd (Alternative condition): a single-rule operation, the as same as in F-1st, but it also requires selection among rules, such as selecting a color or shape rule-response relation. (**c**) F-3rd (Conflict condition): single-rule operation with conflict that also requires suppressing responses to the other feature simultaneously. (**d**) F-4th (Multiple condition): two-rules operation requiring management of both a main and secondary rule set responding to stimuli. The main rule is the same as that in F-3rd. The secondary rule entails judgment about two-rules relations between color and shape concurrently (the process is represented by the lower part of the figure with the direction of the arrow toward “Enter” in the secondary rule figure). A green square in one rule set represents conflict processing, while rules not respond to in each condition are represented by dotted arrows.

In the present study, children at age 4 showed difficulty in managing multiple rules (F-4th in Fig. 2d) even though all subjects at age 4 answered the rules knowledge questions correctly by pointing to the appropriate stimuli and/or by responding with a button press when asked to do so during the practice phase. This indicates that the majority of children at age 4 showed a remarkable dissociation between their knowledge of the rules and their use of those rules. Such behavioral dissociation was also reported in a previous study in which children at age 3 failed to employ rule alternation, despite demonstrated knowledge of the rules^1^. This knowledge–action dissociation has been attributed to insufficiency in the capacity to control both thought and action, according to CCC theory^1,5^, and by insufficient recruitment of higher levels of consciousness (LOC)^38^ in tasks requiring rule integration into a simple rule system. In the present study, over half of the children at early age 4 failed to demonstrate multiple-rules processing capacity by completely failing either the main or secondary rule task: the correct performance rates for either the main or secondary rule was 0%. Also, 30% of children at late age 4 failed to demonstrate multiple-rules processing, and those who succeeded showed a performance level of just 20%. Thus, the error performance at age 4 was mainly attributed to difficulty in concurrent rule management. Therefore, failure in multiple-rules processing in children at age 4 is likely to reflect a functional immaturity in converting multiple rules into a series of integrated rule processing on the F-4th by involving a higher LOC, in order to simplify the internal processes of thought and action. Indeed, it was previously reported that children under age 4 tend to fail at complex task processing due in part to experimental designs requiring use of an abstract rule to control behavior^10,39,40^. Thus, children at age 4 show insufficiency in concurrent multiple-rules processing suggesting that the F-4th system is probably not yet developed by this age.

In accordance with previous studies, we confirmed that performance in the Simple (F-1st in Fig. 2a) and Alternative (F-2nd in Fig. 2b) conditions continues to improve up to age 5, at which point it approaches the adult level. However, performance in the Conflict condition (F-3rd in Fig. 2c) reaches the adult level by late age 6, which is the same timeframe for the development of multiple-rules processing. This may reflect variation in the developmental trajectory not only in rule processing itself but also in the type of cognitive resources involved. The Simple and Alternative conditions require single-judgment processing by considering just one stimulus feature (color or shape), as shown in Fig. 2a,b. The F-1st and F-2nd is involved in short-term memory, and these system are fully constructed by early age 5, even under conditions that require switching between two sets of rules (F-2nd). By contrast, the Conflict condition (F-3rd) requires not only single-judgment processing by attending to one of two stimulus features (color and shape), but also requires suppressing responses to the other feature (Fig. 2c). Thus, the Conflict condition includes short-term memory and inhibitory control, which requires concurrent management of two cognitive resources. The ability to resolve conflicts during information processing has been reported as a special function of selective attention, which helps children focus on a particular stimulus in the service of task demands in the development of EFs^41^. This conflict resolution is considered important for the developmental trajectory of EFs as they build upon previously developed networks^42,43^, and as the complexity of the rule management system^5^ increases. This ability has been shown to develop markedly until about age 6^44,45^. These studies suggested that immaturity of the ability to resolve conflict, a cognitive resource, might at least partially affect the developmental timing of F-3rd. In addition, since the ability of multiple rules-management processing (F-4th) also approached adults levels at the same time as did F-3rd, we cannot rule out the influence of development on the capacity for concurrent processing of multiple EF components. Therefore, the variation in the trajectory of the rule system’s development may reflect variations in the timing of the development of the capacity for hierarchical rule management. Our findings suggest that the development of hierarchical control in the rule management system reflects an age-related shift from independent single-rule processing to integrated multiple-rules processing, especially up to late age 6.

There was a clear performance difference, suggesting differences in the developmental trajectory, in the Multiple condition evident as a linear improvement from ages 4 to 6. This was especially evident between ages 5 and 6, compared with the other conditions. This might reflect a specific and crucial form of EF development during this time period. We speculate that this specific development might reflect changes in integrative functions for managing multiple EF components. This view accords with previous findings that preschoolers’ EF initially arise as unrelated processes, which become more integrated in the preschool to early-primary school years^16,18,20^. This is supported by longitudinal studies of increasing correlations between EF tasks across the preschool years^46^. It is also consistent with a report that EFs were more strongly related in early-primary school students (age 5–6) than in later-primary school students (age 8–9)^17^. The present task paradigm may be capable of identifying the emergence of integrative operations, which appear to arise between ages 5 and 6.

Additionally, performance improvements in the Multiple task may be at least partially due to an age-related qualitative transition, representing a shift from reactive control entailing reactions to events only as they occur and towards a more proactive control that actively prepares for events^47^. This accords with previous findings of age-related progression of EF evident in qualitative changes between preschool and school-aged children^10,12^. Thus, single-rule operations involved in single-EF component processing progresses up to age 5, while multiple-rule operations, of the sort necessary for the concurrent manipulation of these EF components, progresses to late age 6. It is probable that the rule-management system develops in an age-dependent and bottom-up manner resulting in a hierarchical rule system, and that this underlies the hierarchy of cognitive control in the functional organization of the PFC.

In contrast to development-related improvements in performance, that of processing speed showed no significant progression between ages 4 and 6 in the Multiple condition, while the RTs in the Simple, Alternative, and Conflict conditions gradually improved. However, the RTs were significantly slower than those in adults in all conditions. Previous findings suggest a gradual progression in processing speed, with regular incremental improvement from age 6 onward^48–50^. Also, the development of response efficiency appears to rapidly progress at about age 12^15^ and stabilizes by age 15^49^, with continued progress possible into adolescence^51–53^. Thus, development-related improvements in processing speed have a relatively long trajectory. In the present study, all children were in some stage of developing all their EFs, such that processing speed efficiency had not yet developed significantly.

We hypothesize that the development of multiple EFs is supported and limited by the maturation of the prefrontal cortex, especially with regard to functional development in the rostral prefrontal cortex (rPFC). The present task paradigm required the manipulation of multiple EF components simultaneously, while maintaining awareness of a main goal with concurrent awareness of sub-goals. Similar task operations have shown concomitant bilateral activation in the rPFC^29,54,55^. Thus, the performance of our task paradigm may reflect functional development in the rPFC. To our surprise, children at late age 6 showed levels of performance close to those of adults. We speculate that rPFC function undergoes marked development up to age 6, the point at which multiple-operation failures tend to disappear. This lays the foundation for further development during age 6, culminating in the capacity to process operations requiring multiple EF components. This accords with findings that the rPFC exhibits one of the highest rates of brain growth between ages 5 and 11^56^. Further, correlations between regional cortical thickness and functional activation in the FPC have been observed in age 7^57^. Cognitive function-related brain activation appears to be a major structural driver in increasing synaptic connectivity^58^. Future research is needed to assess whether the emergence or capacity for developing multiple-rules processing is due to plastic changes in localized brain structures such as the rPFC.

The present study established a novel task paradigm capable of assessing the emergence and formation of multiple-rules processing in children according to the features of the stimuli presented. While previous studies have suggested that the capacity for complex tasks does not mature until adolescence or adulthood^11,14,51,52^, the multiple-rules performance in the present study revealed adult-like capacity in relatively young children. Since our task paradigm was quite simple, and the rule and processing method could be taught in a stepwise fashion, even young children were able to perform at higher-than-expected levels. The present paradigm might also contribute to our understanding of specific functional development in developmental disorders such as autism spectrum disorder (ASD) and attention-deficit/hyperactivity disorder (ADHD). It was recently demonstrated that impaired performance of multitasking associated with activation in the rPFC is observed children with ADHD^59–63^ as well as in those with ASD^64^, especially when correlated with the severity of their symptoms in Asperger’s syndrome^65^. Thus, an additional utility of our Multiple-rules paradigm may be its potential for elucidating the contribution of EF to symptomology in children with ADHD and ASD.

## Methods

### Subjects

Informed consent was obtained from young adults and all parents prior to their child’s enrollment in the study. The experimental protocol and the study were approved by the Review Committee on Research with Human Subjects of Nihon Fukushi University (11-02, 14-02). This study was carried out in accordance with the Declaration of Helsinki.

A total of 379 subjects comprising 57 adults (22 women; mean age, 19.3 years; SD, 0.5 years) and 322 children participated in this study. Children aged 4 to 6 years were divided into 6-month intervals comprising six groups, as shown in Table 1, to evaluate the developmental trajectories of their rule management systems.

Child subjects with similar social backgrounds were recruited from pre-school (Mebae Child Development Academy) and private and public kindergartens in the Japanese cities of Osaka and Tokoname. Adult subjects were recruited from among students in their second year of rehabilitation coursework at Nihon Fukushi University. The child subjects had no neural tube defects, genetic syndrome disorders, neurological disorders, severe psychiatric disorders (autism, psychosis, oppositional-defiant disorder), or uncontrolled seizures. Information was derived from questionnaires including the Social Communication Questionnaire (SCQ) and the Attention Deficit Hyperactivity Disorder Rating Scale (ADHD-RS)^66^ completed by the parents, and was used to establish the presence of ASD and ADHD. The Wechsler Intelligence Scales for Children (WISC^67^; Fourth Edition) were used for children over age 5, and the Wechsler Preschool and Primary Scale of Intelligence (WPPSI^68^) was used for children at age 4, to exclude subjects with a full-scale IQ lower than 80, as shown Table 1. Adult subjects were excluded if they showed symptoms of depression, as separately measured by the Self-Rating Depression Scale^69^ and the Beck Depression Inventory II^70^. Subjects with dyschromatopia were excluded from the study. Five 4-year-olds and three 5-year-olds were excluded because they failed to understand part of the task paradigm and/or did not complete the experiment. Two adult subjects were excluded due to high scores on the depression scales. The experiment was completed at each subject’s school.

### Procedure

All stimuli were presented on a 17-inch computer screen placed about 55 cm in front of the subjects. The stimulus sequence for each trial was controlled using E-Prime (Psychological Software Tools, Pittsburgh, PA). The completion of all tasks took about 30–40 min, including brief breaks and a practice period before each task. Before the start of each task, the subjects were given instructions about the response button corresponding to the stimuli used in the practice period. Subjects could repeat the practice trials as many times as needed until it was clear they understood the instructions. We then determined whether the subjects correctly understood the task rules by having the examiner verbally ask the subjects to respond to all stimuli printed on paper, followed by checking their answers more than three times. We excluded data the entire subject from this study if the subject failed to understand all of the task rules during the instruction period.

### Task Paradigm

**Figure 3:**
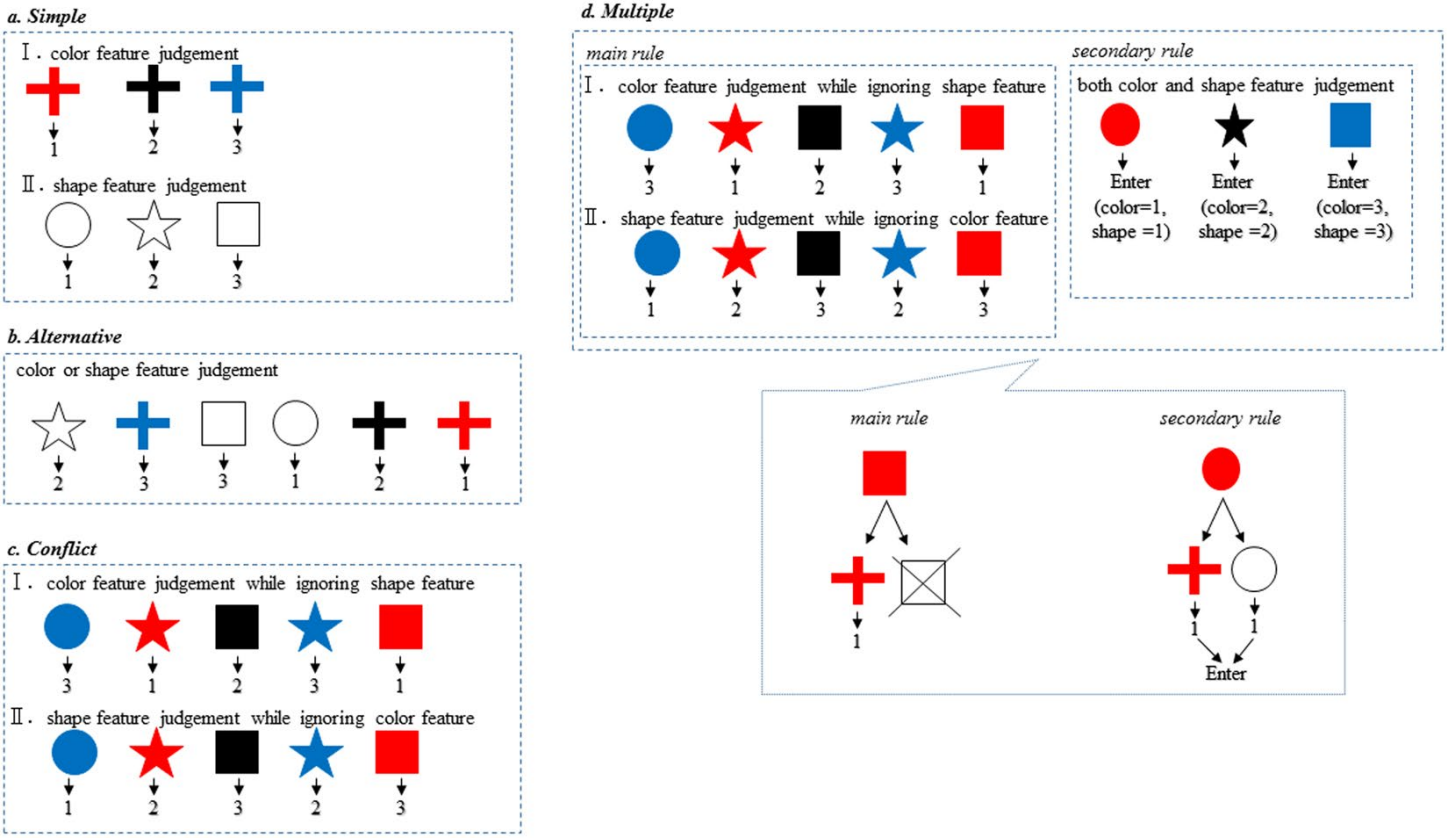
Rule management paradigm There are four task conditions in the rule management paradigm. (**a**) Simple condition, (**b**) Alternative condition, (**c**) Conflict condition, and (**d**) Multiple condition are composed of two color (I) and shape (II) features rules separately. The bottom of d. Multiple condition shows a way of processing main and secondary rule judgments. The rule requires a color feature judgment that requires the subject to ignore the shape feature, while the secondary rule requires judgment about both color and shape features.

We created a “rule management paradigm” comprising four conditional settings (Fig. 3). The first stage of the task (Simple) requires the application of a single rule by responding to one of three stimuli varying by only color or shape. The second stage of the task (Alternative) requires the application of a single rule by alternatively selecting a rule from either the color or shape feature. The third stage of the task (Conflict) requires the application of a single rule based on color or shape to colored shape stimuli (two-featured stimuli), by ignoring one of the two stimuli features. The fourth stage of the task (Multiple) requires the application of multiple rules by selecting between main and secondary rules according to features of the stimuli. In the multiple-rules condition, the main rule is judged by color or shape features and the secondary rule is judged by both color and shape features in the colored shape stimuli (two-featured stimuli). In sum, the Simple and Alternative conditions involve single-rule processing by responding to the color and/or shape feature in the stimuli. The Conflict condition involves single-rule processing by requiring inhibition of another rule responding to one of two conflicting stimuli features. The Multiple condition involves two-rules processing responding to both of two-featured stimuli: the main rule is the same as in the third task condition (Conflict) and the secondary rule requires responses to both features of the stimuli.

**Figure 4:**
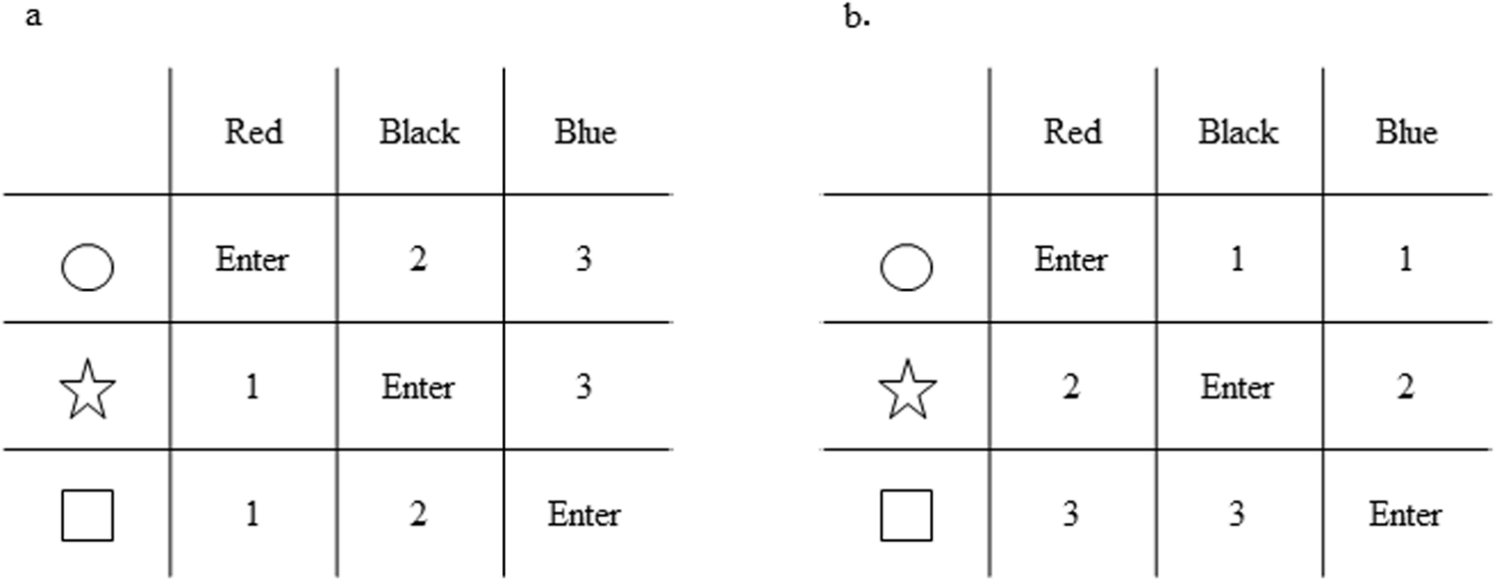
Tables of correct responses in Multiple condition. (**a**) Color rule. Correct responses are determined primarily by the color feature of stimuli (red: button 1, black: button 2, blue: button 3) and secondarily by both color and shape features of stimuli (red-circle, black-star, or blue-square: press “Enter” button). (**b**) Shape rule. Correct responses are determined primarily by the shape feature of stimuli (circle: button 1, star: button 2, square: button 3) and secondarily by both color and shape features of stimuli (red-circle, black-star, or blue-square: press “Enter” button).

As the first stage of the task, each Simple-color and Simple-shape task comprises 15 trials. The subjects must press the corresponding numbered buttons (red = button 1, black = button 2, blue = button 3) in response to the three colors of stimuli in the Simple-color condition; or they must press the corresponding numbered buttons (circle = button 1, star = button 2, square = button 3) in response to the three shapes of stimuli in the Simple-shape condition. As the second stage of the task, the Alternative condition comprises 30 trials in which randomly assigned color and shape stimuli (red or circle = button 1, black or star = button 2, blue or square = button 3). As the third stage of the task, each Conflict-color and Conflict-shape condition comprises 30 trials, using colored shape stimuli. The subjects must respond as in the Simple-color condition to color features of stimuli (red = button 1, black = button 2, blue = button 3) by ignoring the shape feature, or they must respond as in the Simple-shape condition to shape features of stimuli (circle = button 1, star = button 2, square = button 3) by ignoring the color features. As the fourth stage of the task, the Multiple-color and Multiple-shape conditions each comprise 45 trials, using two-featured stimuli. The applicable rules in the Multiple-color and Multiple-shape conditions are used in the Conflict-color and Conflict-shape conditions with the secondary rule requiring a press of a button labelled “Enter” when the response button numbers match in both color and shape rules, such as red-circle, black-star, or blue-square (Fig. 4). In these trial conditions, we specifically instructed the subject to apply the secondary rule by encouraging them to remember the three types of stimuli without providing any more complex explanations about rule application. Thirty-three percent of secondary task stimuli were included in the Multiple condition. Thus, the Multiple-color and Multiple-shape conditions required that the subjects apply either a color or shape criteria as the main rule, while keeping in mind the existence of the three special stimuli as a secondary rule.

**Figure 5:**
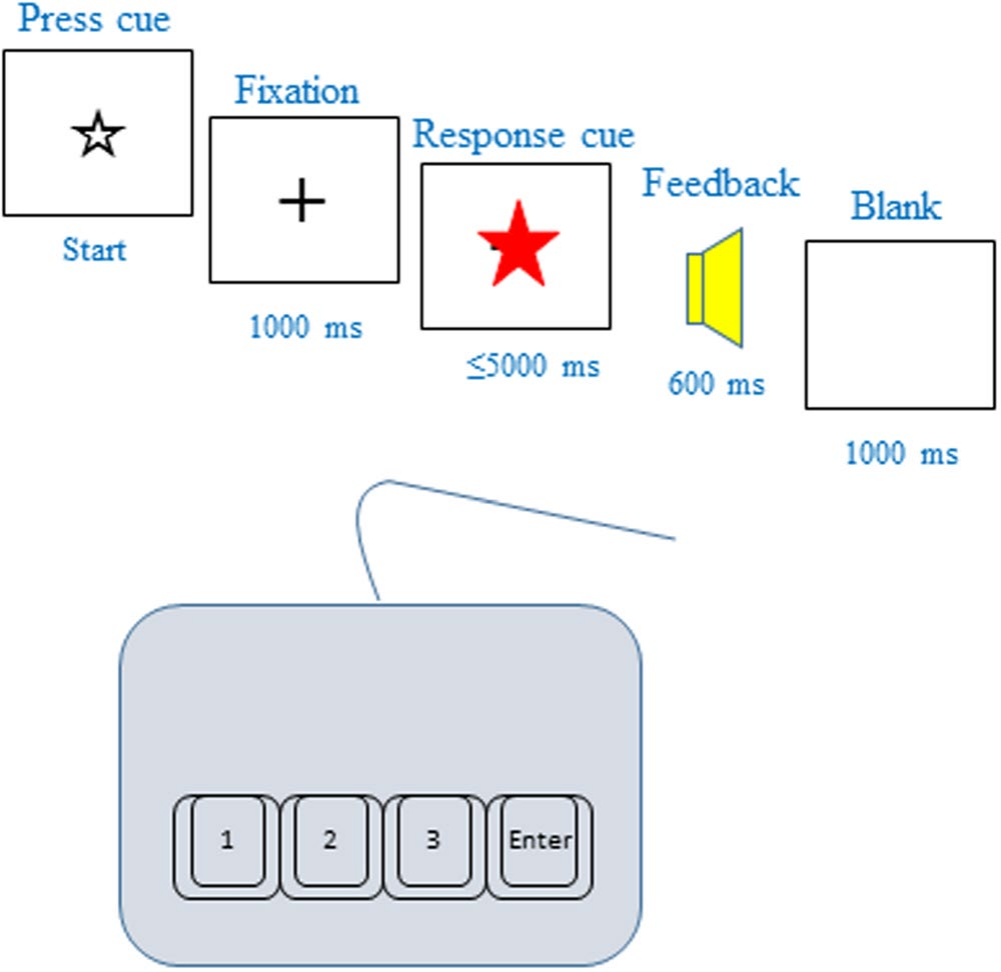
Task sequences. Each stimulus was presented in the center of the screen with a white background. Each trial was started manually by a button press from the experimenter after which a black fixation cross was displayed. A target stimulus was presented 1000 ms after starting and until a response was made, or for up to 5000 ms. Following the response, feedback was provided by a change in the display lasting 600 ms, with auditory feedback comprising either a “woohoo” sound for the correct response or a “beep” sound for an incorrect or missed response. A fixation cross was continuously displayed in the center of the screen, except during presentation of targets or feedback.

Figure 5 shows the task sequence. During task execution, the experimenter sat beside the subject and checked their performance to minimize confusion and ensure task completion. All subjects were instructed to be as fast and accurate as possible. All subjects included in the analysis completed four task conditions in the following order: Simple, Alternative, Conflict, and Multiple. The trial order between the color and shape feature rules in the Simple, Conflict, and Multiple conditions was alternated between successive tasks, e.g., if the color rule was employed first in the Conflict condition, the next Multiple condition also engaged the color judgment rule (e.g., Conflict-color and Conflict-shape was followed by Multiple-color and Multiple-shape). The trial order in task rules between the color and shape features was counterbalanced across all trials for each subject.

### Data analysis

Before the data were analyzed, the correct performance rates for the color and shape feature conditions in the Simple and Conflict conditions were averaged, respectively. For the Multiple condition, this was calculated by the following steps: first, the correct performance rates of the main and secondary tasks were separately calculated in each color and shape feature condition, respectively. Second, the correct performance rates in these main and secondary task were used to calculate a harmonic mean for each feature rule condition. Third, the calculated harmonic means of each feature rule condition were averaged and taken as the correct performance rate for the Multiple task. This calculation was necessary because we needed to determine whether the subject succeeded in concurrent two-rule processing tasks, and to determine whether the subject was able to reach 67% correct performance rate in the Multiple task when the main task was completed successfully but the secondary task was not. The formula for the harmonic mean can be presented as follows, where χ_1_ is correct performance rate on the main task and χ_2_ is correct performance rate on secondary task:

$$H=\frac{2{{\rm{\chi }}}_{{\rm{1}}}{{\rm{\chi }}}_{{\rm{2}}}}{{{\rm{\chi }}}_{{\rm{1}}}+{{\rm{\chi }}}_{{\rm{2}}}}$$

The median RTs per task condition used the speed of correct responses, averaged between color and shape feature rules in the Simple, Conflict, and Multiple conditions, respectively. The data in the Multiple condition were excluded in this analysis if task performance (calculated by harmonic mean) was 0% (Table 2).

Task performances and RTs were analyzed using a one-way MANOVA with seven age groups (early- and late-ages 4, 5, and 6, and adults) as the independent variables, and four task conditions (Simple, Alternative, Conflict, and Multiple) as dependent variables, since the MANOVA permits analysis of several dependent variables. Bonferroni post-hoc analysis was performed when a significant difference was noted. Compliance of residual to a normal distribution was evaluated by Kolmogorov-Smirnov tests and graphically by inspection of histograms of residuals. The effect size (i.e., partial η^2^: η^2^*p*) is also reported to complement the use of significance testing. All statistical analyses were performed using SPSS software (v. 19.0 for Windows; SPSS Japan Inc., Tokyo, Japan). Unless otherwise noted, a 0.05 level of significance was adopted for all statistical analyses.
